# Editorial: Children's health and safety: what we learned from the COVID-19 pandemic and future policy's perspective

**DOI:** 10.3389/fpubh.2023.1220977

**Published:** 2023-06-06

**Authors:** Biagio Solarino, Simona Nicolì, Marcello Benevento, Massimo Zedda, Antonio Oliva

**Affiliations:** ^1^Section of Legal Medicine, Department of Interdisciplinary Medicine, University of Bari, Bari, Italy; ^2^Department of Health Surveillance and Bioethics, Section of Legal Medicine, Fondazione Policlinico A. Gemelli Istituto di Ricovero e Cura a Carattere Scientifico, Università Cattolica del Sacro Cuore, Rome, Italy

**Keywords:** children safety, bioethics in pediatrics, risk management, public health, SARS-Cov2, children

The wellbeing of minors and older adults has always been a public health target. The health and wellbeing of children, enshrined in the Convention on the Rights of the Child (CRC) in 1989, refer to multiple physical, psychological, social, and economic aspects (1). The main factors reventing these rights from being secured are poverty, social isolation, and persistent discrimination.

It is well-known that the health system assumes primary importance in investigating and ensuring the wellbeing of little patients.

The extreme care that has always characterized everything concerning children can also be found in pediatric health issues influencing the sector's workers to deal with thorny implications like patient safety, bioethics, and risk management in a generally multidisciplinary setting.

The scientific literature concerning risk management, patient safety, and bioethics in the pediatric environment requires continuous and updated study by healthcare professionals, in an effort that encounters obstacles not always easy to overcome and in most cases connected with the complexity of the children's health.

We aimed to collect contributions about risk management in pediatric settings concerning bioethical topics and patient safety. Furthermore, in this setting, we also suggest considering the challenges faced by the recent COVID-19 pandemic.

Among the main problems that emerged during the global health emergency, mental disorders in the adolescent population have been addressed by several authors. Kim et al. investigated a possible association between families' economic hardship and mental disorders in Korean adolescents. Anxiety, depressive symptoms, and suicidal ideation seem to be related to the family's economic features, suggesting how economic policies may have a pivotal role in mental health and how it would be helpful to intervene in the health sector by employing online screening and counseling. This last consideration entails consistent ethical and medico-legal challenges to be considered since a complex trade-off between ethical and legal principles must be met to pursue the principle of the best interests of the child (2).

Moreover, Yang et al. investigated a similar issue in the Chinese pediatric population, looking for the risk factors for the increase of the obesity rate. The study identified “grandchild care” as a risk factor for poor pediatric hygiene, i.e., child care provided by older family members, inadequate health insurance policies, and inequalities in socioeconomic factors. This study also offered new prospects for an early intervention involving families, schools, health facilities, and the government, in a multi-level intervention system, with new protections and new social policies. The lockdown imposed by governments has uncovered significant problems relating to hygiene and care of children, highlighting the inadequacy of some parental behaviors and habits and highlighting how the domestic environment often hides many pitfalls and unhealthy behaviors for children.

In this regard, it should be noted that domestic abuse represented a public health concern even before the pandemic, being mainly represented by neglect. However, the risk of interpersonal abuse has globally been increased by the pandemic-related restrictions and thus a frontier in research was to verify if the frequency of minors witnessing interpersonal violence had been increased by the lockdown.

In particular, Focardi et al., an Italian research group analyzed the access in the last 4 years to the Emergency Department of an Italian tertiary hospital due to witnessed violence in the pediatric population. The results highlighted a stationary incidence of the phenomenon compared to previous years. The results are consistent with most studies covering the same period, although in some cases an increase in domestic abuse of minors has been reported (3). This discrepancy could be explained by the protective role played by schools closed during the lockdown, in which educators are generally the first to notice mistreatment. At the same time, promoting education may enhance secondary prevention interventions.

From a clinical perspective, Juul et al. addresses a further challenging issue about surgical waiting lists, which also have implications for pediatric units. This study found that the pandemic has significantly affected the waiting times for elective urological procedures and showed a decrease in the number of those performed. The paper highlights the need for healthcare providers to develop strategies to shorten wait times by ensuring delay-free healthcare.

The access to care of the patients and the safety of the health operators is a critical balance since both clinical and forensic practice hospital environment has different sources of infections, that represent critical hazards if the relatively long persistence of SARS-CoV-2 even on inanimate surfaces is considered. Safety protocols and screening tests of various kinds proved their effectiveness in different hospital contexts (4, 5). Regarding the microbiological risk during the pandemic era, Song et al. found that the transmission of SARS-CoV-2, and thus the infectivity of the virus, varied among different age groups of patients. Furthermore, gastrointestinal symptoms may be the initial presentation of COVID-19 or the only manifestation of the disease in children, and therefore symptoms such as vomiting, diarrhea, and abdominal pain should be considered as part of the screening in pediatric patients.

In this Research Topic, Vetrugno et al. and Bilotta et al. addressed different aspects of the COVID-19 pandemic, highlighting the importance of early screening for SARS-CoV-2 in hospitalized patients and of the vaccination in the pediatric population.

While the first article focuses on preventing the spread of the virus within hospitals, the second study emphasizes the need to protect the health of children and prevent the spread of the virus in the broader community, both providing essential insights into the challenges posed by the pandemic and the need for proactive measures to address these challenges. Bilotta examines the ethical principles of autonomy, beneficence, and non-maleficence in the context of COVID-19 vaccination in children, emphasizing the importance of informed consent and the need for healthcare providers to provide parents with accurate and comprehensive information about the vaccine.

Therefore, the collection of articles on children's health and safety in this Frontiers Research Topic highlights the need for new intervention perspectives and proactive actions for better management of clinical, social, and economic pediatric issues, not forgetting the training gaps of healthcare personnel already operational or still in training. Although the issue of safety in the care of pediatric patients has multiple facets, bioethical issues are still little treated. In conclusion, ensuring a multidisciplinary approach in terms of health care, social interventions, and policy is necessary for the overall wellbeing of children.

## Author contributions

BS and AO contributed to the conception and design of the editorial. SN, MB, and MZ wrote the first draft of the manuscript. All authors contributed to the article and approved the submitted version.
